# Characterisation of Arctic Bacterial Communities in the Air above Svalbard

**DOI:** 10.3390/biology6020029

**Published:** 2017-05-06

**Authors:** Lewis Cuthbertson, Herminia Amores-Arrocha, Lucie A. Malard, Nora Els, Birgit Sattler, David A. Pearce

**Affiliations:** 1 Department of Applied Sciences, Faculty of Health and Life Sciences, University of Northumbria at Newcastle, Ellison Building, Newcastle-upon-Tyne NE1 8ST, UK; lewis.cuthbertson@northumbria.ac.uk (L.C.); ymrhre@hotmail.com (H.A.-A.); lucie.malard@northumbria.ac.uk (L.A.M.); 2 Institute of Ecology, Austrian Polar Research Institute, University of Innsbruck, Technikerstrasse 25, 6020 Innsbruck, Austria; nora.els@uibk.ac.at (N.E.); Birgit.Sattler@uibk.ac.at (B.S.)

**Keywords:** aerobiology, bioaerosol, Arctic, polar, ecology, bacteria, marine, terrestrial, culture dependent, culture independent

## Abstract

Atmospheric dispersal of bacteria is increasingly acknowledged as an important factor influencing bacterial community biodiversity, biogeography and bacteria–human interactions, including those linked to human health. However, knowledge about patterns in microbial aerobiology is still relatively scarce, and this can be attributed, in part, to a lack of consensus on appropriate sampling and analytical methodology. In this study, three different methods were used to investigate aerial biodiversity over Svalbard: impaction, membrane filtration and drop plates. Sites around Svalbard were selected due to their relatively remote location, low human population, geographical location with respect to air movement and the tradition and history of scientific investigation on the archipelago, ensuring the presence of existing research infrastructure. The aerial bacterial biodiversity found was similar to that described in other aerobiological studies from both polar and non-polar environments, with Proteobacteria, Actinobacteria, and Firmicutes being the predominant groups. Twelve different phyla were detected in the air collected above Svalbard, although the diversity was considerably lower than in urban environments elsewhere. However, only 58 of 196 bacterial genera detected were consistently present, suggesting potentially higher levels of heterogeneity. Viable bacteria were present at all sampling locations, showing that living bacteria are ubiquitous in the air around Svalbard. Sampling location influenced the results obtained, as did sampling method. Specifically, impaction with a Sartorius MD8 produced a significantly higher number of viable colony forming units (CFUs) than drop plates alone.

## 1. Introduction

Microbial dispersal in the atmosphere represents a key biological input, directly influencing the gene pool [1]. The dispersal rate of bacteria in the atmosphere has been shown to be directly linked to weather events, such as dust storms, that lift large amounts of microbial matter into the atmosphere [2]. There are two mechanisms by which bacteria are transported through the atmosphere: free floating and attached to larger airborne objects. Free floating bacteria in the atmosphere are unlikely to come into contact with other microorganisms frequently; however, bacteria associated with larger airborne particles could be subject to increased horizontal gene flow [3]. In fact, it is this horizontal gene flow and the abundance of bacteria within the atmosphere which has drawn attention to the environment as a potential source for new antibiotics [4].

Whilst several studies have focused on the movement of bacteria through the atmosphere, the majority of these studies have failed to consider the viability of these colonists upon arrival in their new environments [5]. Microbial matter can be transported through the atmosphere potentially over global scale, allowing long distance colonization. A large number of bacteria also remain viable in the atmosphere for extended periods of time, even under intense selection pressure [6]. These viable microorganisms carry out multiple functions whilst suspended in the atmosphere; these include cloud formation by ice nucleation [7,8], nitrogen processing [9], the degradation of organic carbon-based compounds [10] and photosynthesis [11]. Viable colonists have the potential to interact with microbiomes at the site of deposition in an antagonistic or synergistic way. For example, suspended nitrifying bacteria that are deposited in nutrient poor locations could provide a novel source of nutrients benefitting the ecosystem; conversely, the same mechanism can prove disruptive in other circumstances, causing toxic algal blooms, which can be devastating [12]. Migrating bacteria also pose a potential pathogenic threat to human health, global ecosystem stability [13,14,15] and agriculture due to the homogeneity of modern day crops [16].

Atmospheric bacterial abundance generally ranges from 10^4^ to 10^6^ cells per m^3^ [17], but, this varies throughout the year [18], and can be affected by weather (wind direction, wind speed, temperature, etc.) [19]. Bacterial abundance can decrease by as much as half with increasing altitude, although viable bacteria have been found in the stratosphere at altitudes as high as 7.7 km [2,20]. Bacteria found in the atmosphere are diverse. Airborne bacterial assemblages in both terrestrial and marine environments contain more than 150 genera of bacteria [21,22,23], a level of diversity comparable to other nutrient poor environments such as Antarctic snow, which has been shown to contain in the region of 250 genera of bacteria [24]. Barberán et al. [25] collated over 1000 sampling efforts and found more than 110,000 different species of airborne bacteria in the USA alone. Most bacterial communities in the atmosphere comprise four main phyla: Actinobacteria, Bacteroidetes, Firmicutes, and Proteobacteria, a fact that remains consistent in the atmosphere surrounding both marine and terrestrial habitats [23,26]. However, aerial microbial diversity at genus level is more variable and depends on environmental conditions, such as proximity to agricultural sites, meteorological conditions and season [18,22].

Patterns of diversity in airborne bacterial communities are central to the emerging field of atmospheric biogeography. Indeed, until relatively recently whether microbial biogeography existed in the atmosphere at all was contentious [27]. However, an increasing number of studies have shown the inter-continental dispersal of bacteria across continents separated by both political (Europe and Asia) and geographical (North America and Asia) borders [25,28]. Furthermore, distinct geographical features give rise to distinct airborne microbial communities, for example marine coastal communities are different to continental terrestrial ones [25,29]. Despite these findings, atmospheric biogeography has received little attention as the atmosphere is considered a transport route rather than a stable habitat [30]. The development of aerobiology as a field and improved techniques should help understand whether at the ecological level, microbes interact and evolve within the atmosphere, as they do in other habitats.

The Arctic can be defined as the area above the Arctic Circle. The Norwegian Arctic archipelago of Svalbard is one of the northernmost inhabited locations in the world at 79° N. Svalbard is characterised by its remarkably low human population with only 2185 registered Svalbard inhabitants in 2015 [31]. This low population density translates into reduced anthropogenic environmental alterations such as those linked to agriculture. Thus, the Arctic represents an optimal location to study natural patterns of airborne dispersal and its influence shaping natural communities. Aerobiological studies in the Arctic date back as far as the late 1940s [32]. Studies of this nature are sparse between these early efforts and the present, with very few studies taking advantage of novel molecular techniques. To the best of our knowledge, the only recent terrestrial study of bioaerosols (airborne particles of biological origin) in the Arctic was carried out by Harding et al [33], on Ward Hunt Island located in the Canadian high Arctic. Harding et al. found similarities between air and snow communities and those bacterial communities found in the surrounding Arctic Ocean, drawing the conclusion that local sources are the largest contributors which influence bacterial community assemblages. Their study also found organisms not normally associated with the high Canadian Arctic, microbes from other Arctic locations, as well as some Antarctic microorganisms, supporting the theory of long distance atmospheric dispersal. These findings are consistent with those of previous studies that have stated the dominant groups of bacteria in cold ecosystems to be Proteobacteria (alpha, beta and gamma), Firmicutes, Bacteroidetes, and Actinobacteria [34,35]. However, while aerobiological studies in the Arctic are scarce, the number of studies in the Antarctic has increased [1,36]. To this end, a comparative analysis of aerobiological data over the Arctic and the Antarctic will allow the study of bipolar diversity and potentially, the global atmospheric distribution of microbes.

Organisms in the Arctic atmosphere are exposed to extremely low temperatures and hurricane strength winds, seasonal freeze–thaw cycles, extreme exposure to UV and extremely low levels of nutrients. Thus, organisms inhabiting this region are referred to as extremophiles and tend to exploit features such as the ability to form spores, which allow them to survive the harsh conditions. Similar to those microbes inhabiting the Arctic, organisms surviving in the atmosphere also endure extreme temperatures, UV exposure and poor nutrient levels.

Sampling techniques for terrestrial and aquatic microbial ecology studies are highly variable but based on common principles, established and used consistently. In contrast, a wide range of techniques are available to aerobiology, despite the low number of studies in the field. In general, sampling methods involve impaction, impingement, membrane filtration or the drop plate mechanism, the results of which are not directly comparable due to strong methodological biases. Furthermore, the strength of the bias is still unknown, due to the lack of studies comparing different methodologies, although recent efforts have been made towards establishing a standard methodology [37].

Analytical techniques can also vary considerably among studies, compromising comparability even further. To date, most aerobiological studies use colony-forming units (CFU) count per unit volume of air sampled to measure the density of cultivable microorganisms in the atmosphere. These studies report density changes over space, time and varying environmental conditions; however, culture based studies only provide a partial picture of the overall microbial diversity [30]. Culture dependent studies are also biased towards Gram-positive bacteria, while molecular based studies show the opposite trend, with a large proportion of Gram-negative bacteria populating the aerial environment [38]. For this reason, fluorescence microscopy is increasingly used for cell counts and taxonomic identification, combined with molecular techniques such as high throughput sequencing. Temporal, spatial and meteorological variations also lead to differences in the aerial communities identified [39,40], reducing further the ability to describe biogeographical patterns.

Set against this background, in this study, the influence of different sampling techniques, sampling location and total sample volume on the identification of aerial bacterial communities in the Arctic was explored, based on culture dependent and independent analytical methods, thus presenting a preliminary picture of the microbial community in the air over Svalbard.

## 2. Materials and Methods

### 2.1. Site Description

**Figure 1:**
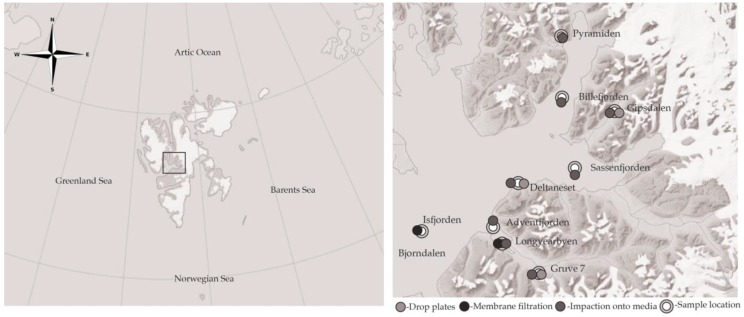
Svalbard location and sampling sites (map adapted with courtesy of the © Norwegian Polar Institute (http://www.npolar.no/no/).

Airborne microbial samples were collected in July 2015 above Svalbard (Figure 1). Svalbard is home to a relatively small human population and plays host to very few mammals. The majority of the human population of Svalbard resides in Longyearbyen; implying that, were samples subject to human influence, it would most likely occur here. The west coast of Svalbard is influenced by the Atlantic Ocean and is affected by warmer currents than the East Coast, oriented towards the Barents Sea.

Samples were collected between 6 and 23 July 2015 above both marine and terrestrial locations using a range of techniques (Table 1). Marine samples were collected aboard the research ship (Viking Explorer) and aboard a zodiac. The terrestrial sites were on the roof of The University Center in Svalbard (UNIS (78°13′ N, 15°39′ E)) located in central Longyearbyen, Mine (Gruve) 7, Deltaneset, Gipsdalen and Bjørndalen; these locations were chosen to represent a large terrestrial geographic range. The marine sites were located in the surrounding fjords at Billefjorden, Isfjorden, Sassenfjorden and Adventfjorden bay (Figure 1).

### 2.2. Meteorological Data

**Figure 2:**
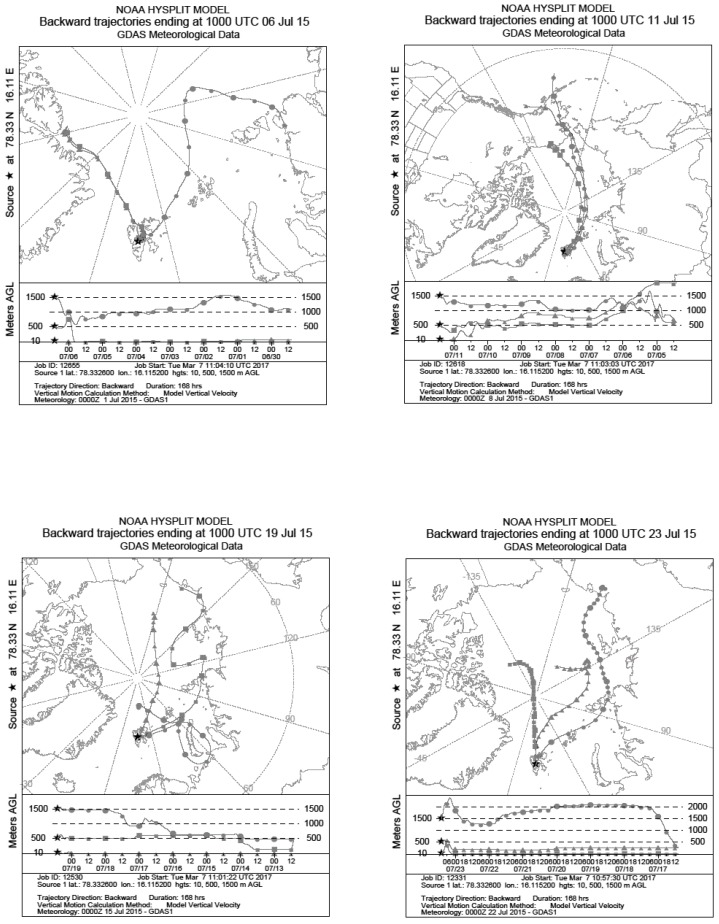
Back trajectory models were calculated using the NOAA Hysplit Model [41]. Three arrival heights were used 10 m (transect marked by triangles), 500 m (transect marked with squares) and 1500 m (transects marked with circles). Sampling location is marked by a black star.

Seven-day back trajectory models were calculated for sampling days where sequencing was carried out at air mass arrival heights of 10 m, 500 m and 1500 m (Figure 2) using National Oceanic and Atmospheric Administration (NOAA) Hysplit Model [41] and the Global Data Assimilation System (GDAS1) archived data file. In general, pockets of air at all altitudes arrived from a northerly (Arctic Ocean) direction, however high altitude air pockets at 1500 m were more easterly influenced than the lower altitudes. On 6 July, the low altitude air masses (10 m, 500 m) were easterly. Temperatures averaged 8 °C across all sampling days with only one precipitation event totalling 0.1 mm occurring on 17 July. Wind speed varied between 10 and 22 km h^−1^ and humidity averaged 67% (Table 2).

### 2.3. Culture Dependent

Drop plates containing R2A media (Sigma-Aldrich, St. Louis, MO, USA) were placed open at Gipsdalen, Mine (Gruve) 7, Deltaneset and Bjørndalen for 15 min; plates were incubated for 10 days at room temperature; following incubation the plates had colony counts and distinct colony counts taken.

Additionally, a portable AirPort MD8 (Sartorius, Göttingen, Germany), comprising a disposable gelatine filter membrane, was used to compare sampling efficiency and cultivability at two flow rates and different sampling volumes. Sampling sites were chosen to compare with terrestrial plate drop sites but also to assess for the differences at marine sites. Terrestrial samples were collected at Mine (Gruve) 7, Deltaneset, Gipsdalen and central Longyearbyen (UNIS roof) and marine samples at Billefjorden, Sassenfjorden and Adventfjorden, respectively. The sampler was used at respective flow rates and durations ranging 30–50 L m^−1^ and 20–80 L m^−1^ on 13, 15, 16 and 17 July 2015. The gelatine filters collected at all sites were placed directly onto the surface of R2A agar plates (Sigma-Aldrich, St. Louis, MO, USA). These plates were then incubated at room temperature for 10 days. Total CFU and distinct colony numbers were counted.

### 2.4. Culture Independent

As gelatine filters are not amenable to culture independent techniques (due to the presence of gelatine), airborne bacteria from both terrestrial and marine sites were collected via membrane filtration. A Welch WOB-L vacuum pump (Welch, Mt. Prospect, IL, USA) was set up at a flow rate of ~20 L m^−1^ connected to Sartorius filtration unit (Göttingen, Germany) containing a 47 mm × 0.2 μm pore size cellulose nitrate membrane filter (GE Healthcare Life Sciences, Chicago, IL, USA).

A marine sample was collected at Isfjorden on the 11 July 2015 with a respective sample duration and volume of 8 h and ~9600 L and the terrestrial sample was taken in central Longeyearbyen (UNIS roof) at the following dates, durations and volumes, respectively: 6 July 2015 for 30 (600 L), 60 (1200 L), 120 (2400 L) and 300 (6000 L) min; 19 July 2015 for 30 (600 L), 60 (1200 L), 120 (2400 L) and 300 (6000 L) min; and 21–24 July 2015 for three days (~86,000 L) continuously (Table 1).

The cellulose nitrate membrane filters were sent to MrDNA (MrDRNA, Shallowater, TX, USA) for extraction and sequencing. DNA was extracted from samples using the MoBio PowerSoil kit (MoBio, Vancouver, BC, Canada) following the manufacturer’s protocol with an additional 1 min of bead beating to account for the filter paper. Extracted samples were then amplified using 16S rRNA universal primers 27Fmod (AGRGTTTGATCMTGGCTCAG) and 519Rmodbio (GWATTACCGCGGCKGCTG) and barcodes were attached at the 5′ end. A 28-cycle PCR using the HotStarTaq Plus Master Mix Kit (Qiagen, Germantown, MD, USA) was carried out under the following conditions: 94 °C for 3 min, followed by 28 cycles of 94 °C for 30 s, 53 °C for 40 s and 72 °C for 1 min, after which a final elongation step at 72 °C for 5 min was performed. After amplification, PCR products were checked in 2% agarose gel to determine amplification success. Samples were then pooled based on their molecular weight and DNA concentrations, purified and illumina DNA libraries were prepared. Paired end sequencing of the V4 region was then performed on a MiSeq following the manufacturer’s guidelines. The resultant data were analysed using QIIME v1.9.1 [42]. The 776,315 raw sequence reads were quality trimmed and checked for chimeras using USEARCH 6.1 [43], clustered at an identity threshold of 97% and assigned to Operational Taxonomic Units (OTUs) using UCLUST [43] and the Greengenes reference database [44] was used to assign taxonomy. Sequences were then aligned using PyNAST [45] and a phylogenetic tree was built using FastTree [46].

### 2.5. Statistical Analysis

Statistical analyses were performed using PAST [47] to test for differences in means, medians, variances and distributions and MS Excel (2013) to calculate correlation coefficients, the coefficient of variance and produce graphs of the analyses; statistical tests were carried out at an assumed significance of alpha: 0.05. When calculating diversity indices, to avoid statistical bias due to differences in sequencing depth all samples were normalised to a depth of 26,190 reads. Rarefaction curves, diversity indices (Shannon and Simpsons reciprocal), Bray-Curtis OTU and unweighted UniFrac phylogenetic distance metrics, and PCoAs were produced using QIIME [42].

## 3. Results

### 3.1. Culture Dependent

**Figure 3:**
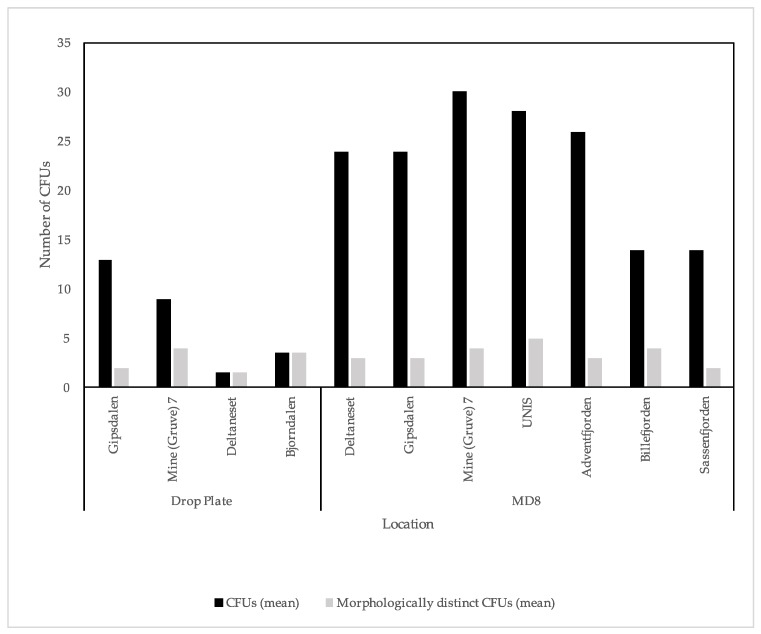
Mean colony-forming units (CFU) and morphologically distinct CFU counts for drop plate and Sartorius MD8 data.

Viable bacteria were found in all of the samples. Clear differences were apparent in the mean CFUs from the two culture dependent methods used (Figure 3). A Kruskal–Wallis test for equal medians of CFUs and morphologically distinct CFUs was undertaken to assess the drop plate replicates, the result did not show significant differences (Kruskal–Wallis plate fall CFU: *p* = 0.095, plate fall morphologically distinct CFUs: *p* = 0.123).

**Figure 4:**
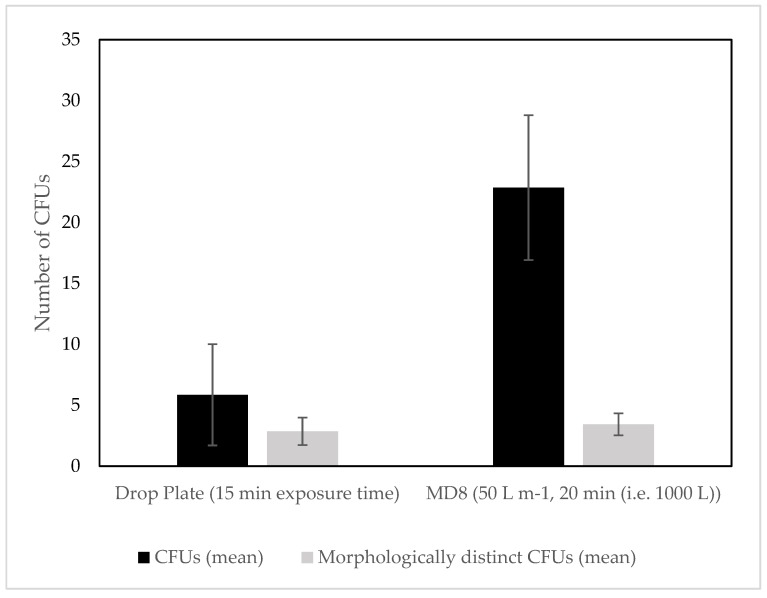
Mean CFUs and mean morphologically distinct CFUs for drop plates and 1000 L MD8 samples.

Comparing the differences in drop plate and MD8 results for the locations where data were available for both methods, the MD8 showed much higher CFU yields, although this difference is not obvious when looking at the number of morphologically distinct CFUs i.e., CFUs of different appearance (Figure 3). Statistical analyses show a significant difference of mean CFUs sampled at the same location using different methods (*p* < 0.05); no differences in variances, medians or coefficient of variations, but a significant difference in equality of distributions (Kolmogov–Smirnov: *p* < 0.05). For the morphologically distinct CFUs, however, there were no significant differences for any of the mentioned parameters. When looking at the overall variance and efficiency of both culture dependent methods, only considering the MD8 samples collected at 50 L m^−1^ for 20 min i.e., 1000 L sampling volume (Figure 4), there were obvious differences in the mean CFUs, but not for morphologically distinct CFUs. An independent *t*-test comparing the two methods showed a significant difference in the mean CFUs from drop plate and MD8 samples (*p* < 0.001), no significant differences in variances, but with significant differences in coefficients of variation (*p* < 0.005), medians (Mann–Whitney U *p* ≤ 0.001) and distributions (*p* < 0.001). Looking at the statistical analysis of the morphologically distinct CFUs, there was no significant difference in the means from drop plates and the MD8 (*p* > 0.05), with no significant differences in variances, medians, distributions, or coefficients of variation. These results show that there is a significant difference between the two methods, the MD8 yielding a larger number of CFUs.

**Figure 5:**
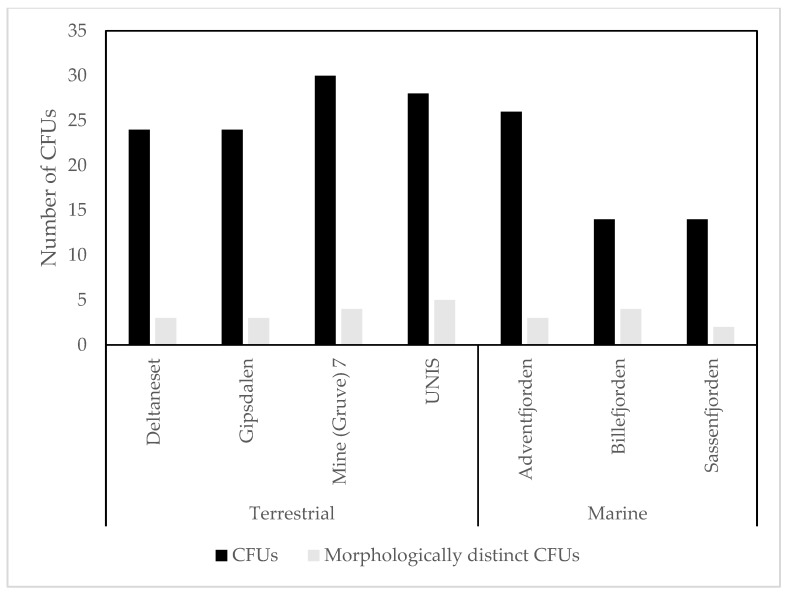
Counts of total (black) and morphologically distinct (grey) CFUs in each sample separated by environment (terrestrial and marine).

Comparing MD8 results for terrestrial and marine samples, the mean CFUs were lower at marine sites than terrestrial sites (Figure 5). Statistical analysis showed no significant difference in CFUs for marine and terrestrial sites (*p* = 0.070). This also held for the morphologically distinct CFUs, there was no significant difference for either of the mentioned parameters between terrestrial and marine sites.

**Figure 6:**
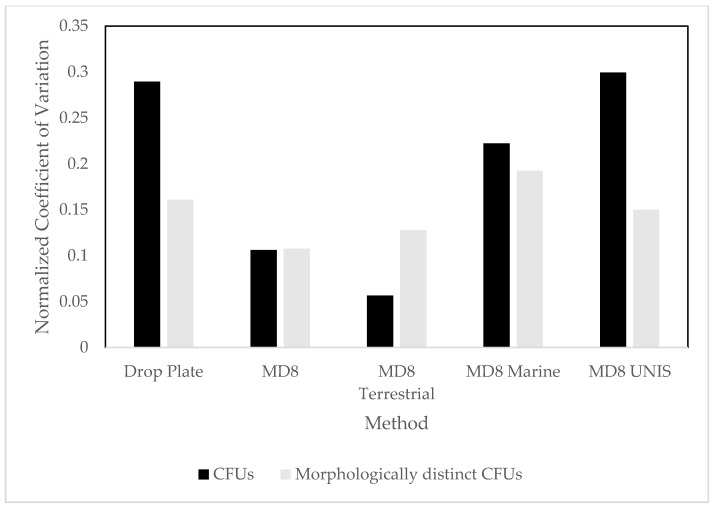
Normalised coefficient of variation (a ratio of mean and standard deviation without unit, to compare different scales, here normalised to account for small sample size).

To account for the variable scales across the samples, the normalised coefficient of variation was used, and this showed the highest variation in the CFUs in the drop plate and MD8 samples collected at UNIS. In the MD8 marine sample the variability of morphologically distinct CFUs was the highest. The CFUs at the terrestrial sites varied the least (Figure 6).

**Figure 7:**
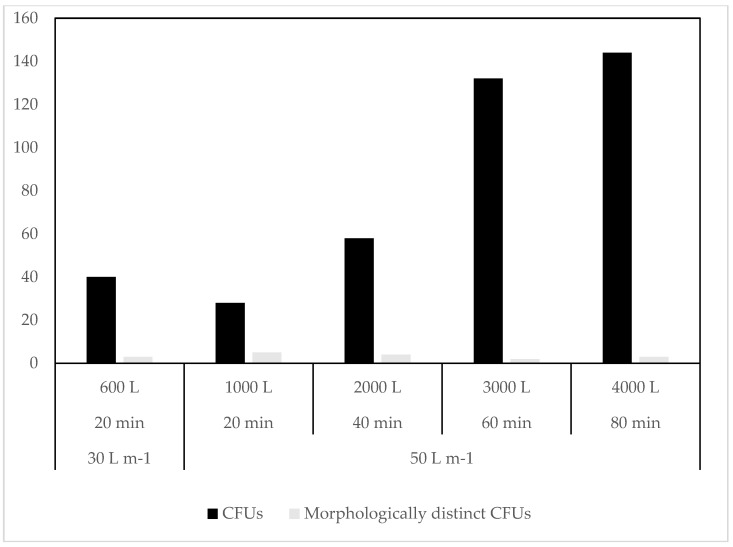
MD8 samples with increasing sample volume at UNIS.

**Figure 8:**
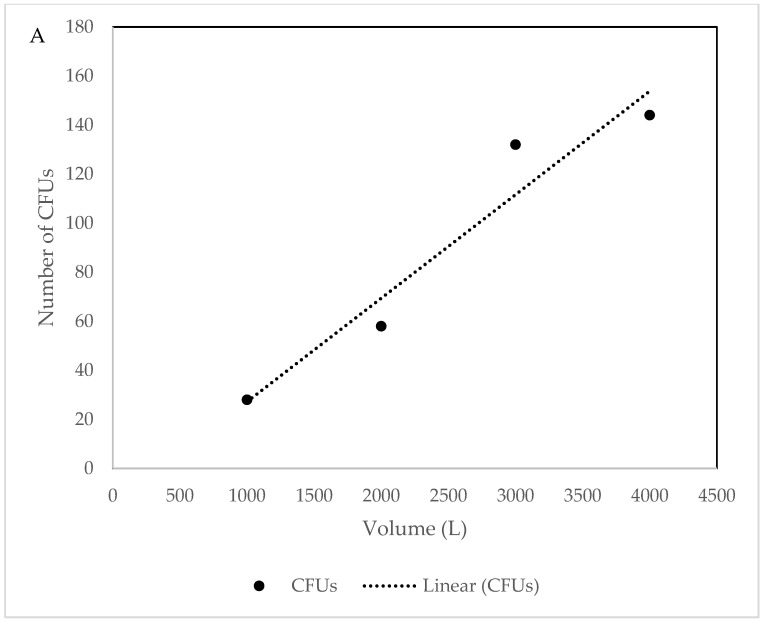

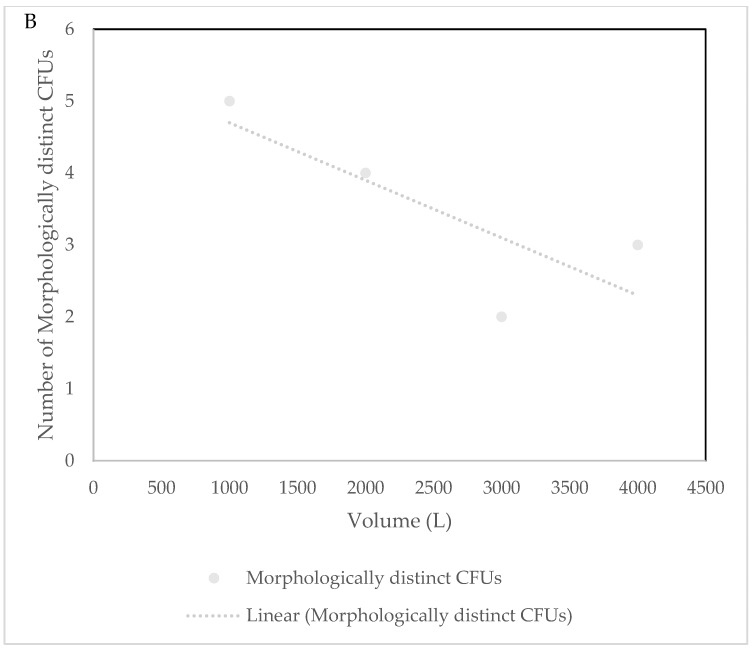
(**A**) CFUs sampled against total volume of air (excluding 30 L m^−1^ sample). (**B**) CFUs sampled against total volume of air (all samples).

At UNIS, where volume and flow rate were varied, a clear trend of increasing CFUs with increasing volume was evident (Figure 7). The 30 L m^−1^ flow rate sample, however, had slightly higher CFUs, despite lower volume. There was a clear correlation between CFUs and the sampled volume of air (*R*^2^ = 0.933, Figure 8A), not including the 30 L m^−1^ sample, and still a very high positive correlation of (*R*^2^ = 0.906) when this sample was included. For the morphologically distinct CFUs, there was a slight negative correlation (*R*^2^ = −0.256) in number of CFUs with increasing volume (Figure 8B), leaving out the exceptional value of 30 L m^−1^ showed a considerable negative correlation (*R*^2^ = −0.640).

### 3.2. Culture Independent

#### 3.2.1. Bacterial Diversity

**Figure 9:**
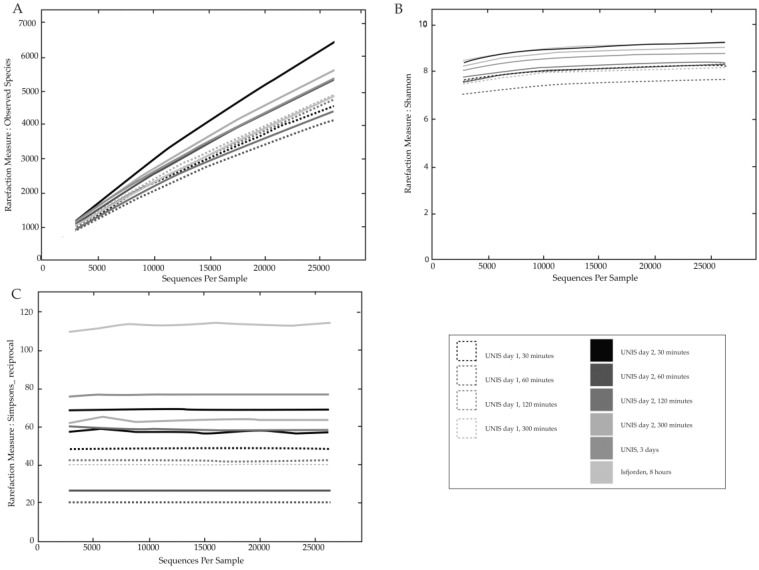
α-Diversity measures: (**A**) Rarefaction curves for observed species; (**B**) Shannon index; and (**C**) Simpsons reciprocal index.

Targeted amplicon sequencing of the 16S rRNA V4 region resulted in 776,315 total reads across the 10 samples, which were then quality filtered and checked for chimeras leaving 685,583 reads. The range of reads per sample ranged from 32,651 (recorded in the 60 min sample from 6 July) to 145,488 reads (recordedin the 30 min sample collected on 19 July). Samples were then rarefied to 26,190 reads (lower than the smallest sample); rarefied samples averaged 5015 OTUs (range 4143–6402). Rarefaction curves for all of the normalised samples did not reach asymptote suggesting the full extent of the diversity present was not reached for all samples (Figure 9A). The Shannon diversity index, a proxy for richness and evenness, was similar in all samples (Figure 9B). The results showed that all samples shared similar levels of diversity (Shannon index range 7.66–9.28); the most diverse sample based on the Shannon index was the 30 min sample taken on Day 2 whilst the least diverse sample based on this metric was the 60 min sample on Day 1. The dominance Simpsons reciprocal index showed a larger difference in the degree of diversity between samples, showing the marine sample to be the most diverse with a Simpsons reciprocal value of 114.13 whilst the lowest diversity was seen again in the Day 1 60 min sample with a value of 20.35 (Figure 9C).

**Figure 10:**
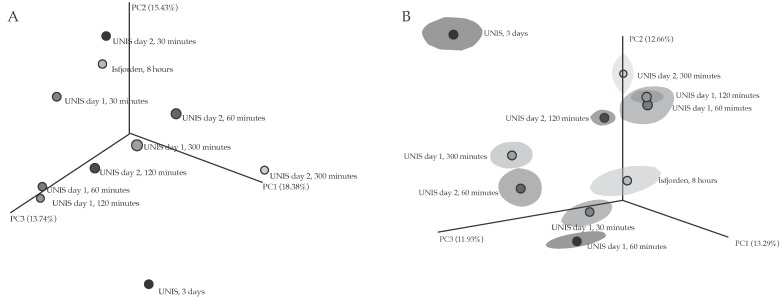
Jackknifed β-diversity metrics: (**A**) Bray-Curtis Index; and (**B**) Unweighted UniFrac.

The differences in OTU diversity between the communities was measured using the Bray-Curtis dissimilarity index (Figure 10A) and an un-weighted UniFrac was used to estimate the phylogenetic distance between different communities (Figure 10B), the variation across all PCoA axis was low. Both metrics showed no distinct pattern between sampling days; however, sampling location did have an effect and different sampling durations showed minor clustering between the 60 and 120 min durations on Day 1. All samples reported differing levels of richness and evenness (Figure 10A) and showed considerable phylogenetic distances with the greatest distance in the three-day sample (Figure 10B).

#### 3.2.2. Taxonomy

**Figure 11:**
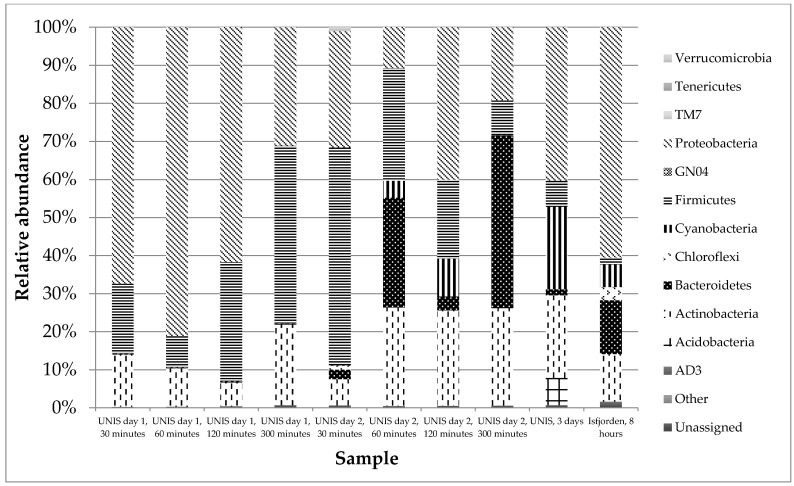
Phyla level relative abundances (%) of bacteria in all culture independent samples.

Twelve phyla in total were detected within the samples: Proteobacteria, Firmicutes and Actinobacteria were present in all of the samples at differing but high relative abundances and were the visibly dominant phyla (Figure 11); Bacteroidetes, Chloroflexi and Cyanobacteria were also present in all samples; and Cyanobacteria and Bacteroidetes were present in sporadically large relative abundances, however, in general, these three phyla were present at <1% (Figure 11).

Proteobacteria, Firmicutes and Actinobacteria represented ~99% of the Day 1 sample set in which there were 10 phyla present in total (Figure 11). Proteobacteria showed the largest total and range of relative abundance on this sampling day. On Day 2, there were 12 phyla present. Proteobacteria, Firmicutes and Actinobacteria remained the three key phyla at a total average relative abundance of 75%. The decrease in relative abundance from Day 1 was mirrored in the 60 and 300 min duration samples by an increase in the average relative abundance of Bacteroidetes. The three-day sample contained 10 phyla as for Day 1, but showed similar phyla and relative abundances to the 60 min sample on Day 2. Acidobacteria were present at 6% in this sample, but they were present at <1% relative abundance in all other samples. The marine sample collected at Isfjorden contained 10 distinct phyla, the same number present in the Day 1 and three-day sample.

There were 196 genera in total, 58 of which were present in all samples. The marine sample taken at Isfjorden contained the highest number of distinct genera with 148, whilst the terrestrial 120 min sample on Day 2 contained the lowest number of genera at 100. On Day 1, the average number of genera present was 190 whilst on Day 2 the number dropped to 113. In the three-day sample at UNIS there were 130 genera, more than in any of the other eight samples collected at that location. *Pseudomonas*, *Staphylococcus*, *Propionibacterium*, *Delftia* and *Corynebacterium* spp. made up the five most relatively abundant genera. *Pseudomonas* was the most common and relatively abundant genera representing 18% of the full sample set, however, they were only the most abundant genera in the Day 1, 30 min sample. *Pseudomonas, Acinetobacter, Corynebacterium, Staphylococcus, Deltia, Cloacibacterium, Arthrobacter, Sphingomonas, Alcanivorax, Comamonas, Streptomyces* and *Brevibacterium* spp. were all regularly present in the top 10 most abundant genera in each sample (Table 3). Members of the order Lactobacillales and Alcaligenes were both present in all four Day 1 samples but just one Day 2 samples whilst *Microbacterium*, a genus of the Microbacteriaceae family and a member of the Intrasporangiaceae family were present in all four Day 2 samples but just one Day 1 sample. There were 15 genera specific to Day 1 and 17 specific to Day 2. The three-day sample recorded six genera specific to that sample. The marine sample recorded the highest number of sample specific genera with 17.

## 4. Discussion

### 4.1. Culture Dependent

All culture dependent samples recorded growth, showing that viable microbes are common in the atmosphere, at both terrestrial and marine locations around Svalbard. The number of viable bacteria measured in the air was considerably lower than the number measured in other environments e.g., surface ice and cryoconite holes, where the number of CFU can be up to tenfold higher using the same media [48]. These results suggest that the atmosphere represents an extremely selective environment, although it is worth noting that only 0.2–2% of the culturable bacteria in the atmosphere are typically recovered by culture dependent studies [49,50]. Previous studies using both drop plates and impaction based techniques similar to the Sartorius MD8 have shown impaction to consistently produce more CFUs [51], in line with this, the impaction method used here produced significantly higher CFUs overall. At locations where both methods were employed, drop plates underestimated the number of viable microbes compared to impaction, likely because a much higher volume of air is being actively directed onto the collection plate using impaction. There was no significant (*p* > 0.05) difference in the number of morphologically distinct CFUs produced by the two methods, and none of them showed any particular bias for specific taxonomic groups on R2A. Replicates taken for drop plate samples showed the technique to be robust with no significant difference across the samples. Generally, marine studies tend to present more CFUs than terrestrial samples [52]. Despite this, there were no significant differences between these two environments; although when normalising coefficients of variations in the sample a clear difference was visible. In our case, the highest number of viable bacteria was in the samples taken at UNIS, consistent with the diversity of activity in that location.

The number of cultivable bacteria increased with the increase in sample volume when using the MD8. This contradicts previous studies which showed no effect of sample volume on total CFU counts [53]. In addition, decreasing the flow rate from 50 L m^−1^ to 30 L m^−1^ increased the number of cultivable bacteria recovered, possibly due to the decreased impact stress placed on captured bacteria [54].

Whilst culture dependent studies provide useful information about the proportion of viable bacteria in the atmosphere, it is generally considered that only around 1% of the total bacteria present in the atmosphere are culturable [55]. Dormancy may represent an important survival mechanism for bacteria in the atmosphere; therefore, a considerably larger proportion of viable non-culturable bacteria (VBNC) would also be expected and may have been overlooked in previous studies based on culture techniques alone. The reliance on CFU counts and inability to describe VBNC bacteria limits the value of culture dependent techniques from an ecological perspective.

### 4.2. Culture Independent

Culture independent studies using sequencing can provide more information about the diversity and taxonomic composition within an environment. Despite the ability of culture independent studies to generate useful information, they also have major drawbacks, as they contain little information about the viability of the bacteria in the environment. Thus, combining both culture dependent and culture independent methods, provides a better insight into both the structure and viability of bacterial communities.

Previous research on bacteria in the atmosphere outside the Arctic has linked temporal and spatial variation to changes in the diversity and abundance [56]. Despite these factors impacting bacterial communities in other Arctic ecosystems such as soil [57], there are no studies to date which investigate these patterns in the atmosphere in this region. Temporal variation (sampling day) did appear to have an effect on community structure, as the composition of the dominant Day 1 phyla was clearly different to that on the other three sampling days. Spatial variation (marine and terrestrial) also appeared to have an effect, although this was less pronounced than the temporal variation, as the dominant phyla present in both the marine and terrestrial samples was consistent. Our results suggest that day of sampling (temporal) is more important than location (spatial) with regards to sample diversity most likely due to changes in meteorological conditions such as wind direction which appeared to produce distinct communities at the phylum level (Figure 11). Duration also appeared to have an effect on the taxonomy of the communities, because whilst the dominant groups of phyla remained constant, the relative abundances varied considerably with changing duration. Although this variation could relate to confounding factors such as the time of day the samples were taken and the duration of sampling.

#### 4.2.1. Diversity

Samples did not cluster into distinct groups based on OTU or phylogenetic relationships, showing no direct link between diversity and sampling duration, location or day (Figure 10A,B). On Day 1, 60 and 120 min samples clustered based on both relationships, likely due to the samples sharing similar relative abundances of *Delftia*, *Ralstonia* and *Pseudomonas*. A higher Simpson reciprocal value was seen on the third sampling day (76.61) taken at UNIS, suggesting that sampling for a longer duration increases the diversity of bacteria captured. The marine sample was considerably more diverse than the terrestrial samples when taking into account dominance (Figure 9C), further supporting the idea that the distinct geographical features of marine coastal locations when compared to terrestrial ones give rise to more varied communities [25,29]. Meteorological conditions such as wind speed, humidity and pressure are known to directly impact community structure [19]; however, during our study, these conditions remained relatively constant, which could explain the similar levels of diversity of the samples shown by the Shannon index (Figure 9B).

#### 4.2.2. Taxonomy

A maximum of 12 phyla were found in air samples from Svalbard; however, the number of phyla varied among samples. The pattern found on Day 1 was the most distinct with three phyla dominating. The distinctiveness of the pattern on Day 1 was likely due to easterly winds from a low altitude air mass leading up to and during this sampling occasion. During the other sampling days, the predominant wind had a main westerly component. The 12 phyla could be separated into two groups: the primary phyla Proteobacteria, Firmicutes and Actinobacteria; and the remaining phyla that were present in sporadic relative abundances. This pattern is consistent with previous studies in cold ecosystems [23,26,34,35] and with bioaerosols from a range of environments [1,18,40,58]. Bacteroidetes could be considered a primary phyla, as they were present in considerable relative abundance in all of the samples apart from on Day 1, suggesting their source is to the east of Svalbard due to the back trajectory of the prevailing wind direction. The primary phyla are probably well adapted to atmospheric life, e.g., Firmicutes are well known for their ability to form spores in low nutrient conditions [59]. Actinobacteria have a higher GC content than other bacteria [60], which is a useful defence against the increased UV exposure faced by bioaerosols, and Proteobacteria are known to fill a multitude of niches due to the metabolic diversity of the group [61]. The number of phyla occurring in the air above Svalbard is considerably lower than that described for urban environments, with studies reporting the number of distinct phyla present to be as high as 38 [58], likely due to differences in the environments. It is notable that *Deinococcus* sp. were not present in any of the samples, a group of bacteria normally associated with atmospheric studies, both in the Artic and elsewhere [33,56]. *Bacillus* sp. were responsible for a large proportion of the Firmicutes present in the sample, the source of which in the terrestrial samples was likely the surrounding soil [39]. There also appeared to be a relationship between the Actinobacteria and the Pseudomonadales whereby as the relative abundance of one increased, the other decreased as has been found previously [62]. Interestingly there was a spike of Acidobacteria in the three-day sample which could suggest this phyla is best adapted to survive the stress of desiccation caused by sampling for longer periods.

At the genus level, the patterns were much less distinct. Of the 196 genera, only 58 were present in all samples. The five most relatively abundant genera (*Pseudomonas, Staphylococcus, Propionibacterium, Delftia* and *Corynebacterium* spp.) are all either polar associated or ubiquitous. *Delftia* sp. have been described at multiple Arctic locations including Svalbard and Greenland where they are associated with surface ice [63,64] whilst *Propionibacterium* sp. are typically associated with marine sediment in the Arctic Ocean [65,66]. *Pseudomonas* sp. are ubiquitous and present in almost all polar studies, however, on Svalbard they are mainly described from fjords [67]. Indeed, a new psychrophilic species of *Pseudomonas* was recently described from the same region [68]. *Corynebacterium* sp. have previously been found in soils from the Canadian high Arctic [69]. *Staphylococcus* sp. were frequently present, but are not routinely described in environmental Arctic studies and could be human or animal associated. *Acinetobacter* sp. are also commonly found in the top 10 most relatively abundant bacteria in all the locations. *Acinetobacter* sp. have been found in glacial snow and ice in mountainous locations outside the Artic [70], however, are mainly associated with marine environments such as fjords in Svalbard [67]. *Alcanivorax* sp. and members of the Oxalobacteraceae family were also common, they appeared on the days dominated by easterly winds and did not appear on the day dominated by westerly winds [71,72]. Members of the Oxalobacteraceae family have also been described in Arctic soils [73]. *Polaribacter* sp., a bacterium associated with polar sea ice, was present in the marine sample suggesting that the Arctic Ocean provides a source of bacteria to the atmosphere. Many of the regularly occurring marine psychrotrophs, included in the *Pseudomonas, Acinetobacter, Alcanivorax, Psychorbacter* genera and members of the Oxalobacteraceae family are associated with the degradation of hydrocarbons in the Arctic [74], which are abundant in Svalbard fjords. The number of distinct phyla recovered on Svalbard (12) was higher than the number recovered over Ward Hunt Island (WHI) in the Canadian high Arctic [33] where six distinct phyla were found. Several of the 14 genera described in the air on Ward Hunt Island (WHI) were also present on Svalbard, including *Cytophagales, Lactobacillus, Staphylococcus, Janthinobacterium, Pseudomonas* and *Polaromonas*, which were mentioned but excluded as a chimeric sequence in that study. Bipolar comparisons also give an insight into both long-range transport and biogeography. Thus, Pearce et al. [1] described the presence of *Acidovorax, Acinetobacter, Cloacibacterium, Pseudomonas* and *Sphingomonas* at Halley station in Antarctica, all of which were present at varying relative abundances in Svalbard air.

## 5. Conclusions

Abundant viable bacteria from a reduced range of bacterial phyla were found in the air above Svalbard. The number of viable colonies (CFUs) present was related to variation in both sampling technique (with the concentration of viable CFUs being higher when using a Sartorius MD8 when compared to drop plates) and sample volume, with increasing volume increasing total viable CFUs. The communities described were fairly homogeneous across sites, suggesting a distinct aerial community above Svalbard. Airborne bacterial abundance was lower than that described from other Arctic environments, such as the soil or the ice surface. The most relatively abundant taxa were polar associated, suggesting that the largest input into the atmosphere on Svalbard was of local origin. The overall diversity of the phyla present in the air above Svalbard was less diverse than in other locations such as urban environments, but was similar to that described previously in the Arctic on WHI. The key phyla remained consistent across studies.

Further studies using metatranscriptomics would provide a deeper insight into the ecological role and metabolic activity of airborne bacteria, and potentially their ability to sustain activity, colonize and alter the environment at their final destination. Future studies investigating the biodiversity of the airborne microbes present in the Arctic will provide an insight as to whether an indigenous Arctic community exists.

## Figures and Tables

**Table 1:** Summary of sample locations and regimes.

Sample Location	Environment	Sampling Mechanism	Date	Flow Rate (L m^−1^)	Duration (min)
Bjørndalen	Terrestrial	Drop plates	13 July 2015	-	15
Deltaneset	Terrestrial	Impaction onto media	13 July 2015	50	20
		Drop plates	13 July 2015	-	15
Gipsdalen	Terrestrial	Impaction onto media	13 July 2015	50	20
		Drop plates	13 July 2015	-	15
Longyearbyen	Terrestrial	Impaction onto media	16 July 2015	30, 50	20, 40, 60, 80
		Membrane filtration	06, 19, 21–23 July 2015	~20	30, 60, 120, 300, 3 days
Mine (Gruve) 7	Terrestrial	Impaction onto media	13 July 2015	50	20
		Drop plates	13 July 2015	-	15
Adventfjorden	Marine	Impaction onto media	17 July 2015	50	20
Billefjorden	Marine	Impaction onto media	17 July 2015	50	20
Isfjorden	Marine	Membrane filtration	11 July 2015	~20	480
Sassenfjorden	Marine	Impaction onto media	17 July 2015	50	20

**Table 2:** Meteorological conditions on sampling days at Svalbard airport (The Weather Company (Atlanta, GA, USA)).

	06 July 2015	11 July 2015	13 July 2015	16 July 2015	17 July 2015	19 July 2015	21 July 2015	22 July 2015	23 July 2015	21–23 July 2015 (Average)	Average across All Sampling Days
Average temperature (°C)	8	10	8	6	8	8	10	8	6	8	8
Total precipitation (mm)	0	0	0	0	0.1	0	0	0	0	0	0
Average wind speed (km h^−1^)	13	20	10	20	14	22	18	12	12	14	16
Average humidity (%)	63	75	90	57	68	59	65	68	61	65	67
Pressure (hPa)	1025	1022	1019	1009	1013	1015	1015	1013	1006	1012	1016

**Table 3:** Top 10 most abundant OTUs in each sample labelled at their highest resolution.

**UNIS Day 1, 30 min**		**UNIS Day 1, 60 min**		**UNIS Day 1, 120 min**		**UNIS Day 1, 300 min**	
OTU assignment	Relative abundance	OTU assignment	Relative abundance	OTU assignment	Relative abundance	OTU assignment	Relative abundance
*Pseudomonas*	38%	Pseudomonadaceae	39%	Pseudomonadaceae	31%	*Corynebacterium*	17%
*Acinetobacter*	13%	*Corynebacterium*	22%	*Staphylococcus*	22%	Unassigned	16%
Bacillales	10%	*Pseudomonas*	13%	Unassigned	12%	*Acinetobacter*	14%
*Corynebacterium*	9%	Unassigned	8%	Streptophyta	9%	Gaiellaceae	14%
*Staphylococcus*	8%	*Micrococcus*	7%	Bacillales	9%	Pseudomonadaceae	9%
Pseudomonadaceae	8%	Bacillales	3%	*Pseudomonas*	6%	Pseudomonadaceae	9%
*Delftia*	7%	Gammaproteobacteria	2%	Pseudomonadaceae	3%	*Staphylococcus*	7%
*Propionibacterium*	5%	Gaiellaceae	2%	Burkholderiales	3%	Bacillales	5%
Unassigned	0%	Nocardioidaceae	1%	Acetobacteraceae	2%	Acetobacteraceae	3%
*Cloacibacterium*	0%	*Acinetobacter*	0%	*Sphingomonas*	1%	Alcaligenaceae	2%
**UNIS Day 2, 30 min**		**UNIS Day 2, 60 min**		**UNIS Day 2, 120 min**		**UNIS Day 2, 300 min**	
OTU assignment	Relative abundance	OTU assignment	Relative abundance	OTU assignment	Relative abundance	OTU assignment	Relative abundance
Unassigned	49%	Oxalobacteraceae	27%	Oxalobacteraceae	26%	Comamonadaceae	36%
*Corynebacterium*	18%	*Acinetobacter*	15%	*Staphylococcus*	20%	Candidate division TM7	24%
Alcaligenaceae	7%	*Arthrobacter*	14%	*Arthrobacter*	11%	*Alcanivorax*	6%
*Staphylococcus*	6%	*Corynebacterium*	11%	*Brevibacterium*	10%	Bacillaceae	5%
*Arthrobacter*	2%	Alcaligenaceae	11%	Candidate division TM7	9%	*Enterococcus*	3%
Comamonadaceae	2%	Nocardioidaceae	4%	iii1-15	8%	Gaiellaceae	3%
*Acinetobacter*	2%	Unassigned	3%	*Corynebacterium*	4%	*Brevibacterium*	3%
Candidate division TM7	1%	*Streptomyces*	3%	Comamonadaceae	3%	*Comamonas*	3%
Gaiellaceae	1%	*Staphylococcus*	2%	Weeksellaceae	3%	*Staphylococcus*	2%
*Pseudomonas*	1%	*Cloacibacterium*	2%	*Comamonas*	1%	*Acidovorax*	2%
**Isfjorden, 8 h**		**UNIS, 3 Day**					
OTU assignment	Relative abundance	OTU assignment	Relative abundance				
Oxalobacteraceae	39%	Oxalobacteraceae	22%				
*Bacteroides*	10%	*Alcanivorax*	14%				
iii1-15	6%	Burkholderiales	7%				
*Delftia*	5%	*Corynebacterium*	6%				
*Burkholderia*	4%	oc28	6%				
*Achromobacter*	4%	Candidatus *Aquiluna*	5%				
Caulobacteraceae	3%	Bacillaceae	5%				
Comamonadaceae	3%	Microbacteriaceae	5%				
Staphylococcaceae	2%	*Streptomyces*	4%				
Burkholderiales	2%	iii1-15	4%				
